# An Economic Assessment Model of Rural and Remote Satellite Hemodialysis Units

**DOI:** 10.1371/journal.pone.0135587

**Published:** 2015-08-18

**Authors:** Thomas W. Ferguson, James Zacharias, Simon R. Walker, David Collister, Claudio Rigatto, Navdeep Tangri, Paul Komenda

**Affiliations:** 1 Department of Medicine, Section of Nephrology, University of Manitoba; Winnipeg, Canada; 2 Department of Community Health Sciences, University of Manitoba; Winnipeg, Canada; Department of Transplantation and Renal Medicine, AUSTRALIA

## Abstract

**Background:**

Kidney Failure is epidemic in many remote communities in Canada. In-centre hemodialysis is provided within these settings in satellite hemodialysis units. The key cost drivers of this program have not been fully described. Such information is important in informing the design of programs aimed at optimizing efficiency in providing dialysis and preventative chronic kidney disease care in remote communities.

**Design, Setting, Participants, and Measurements:**

We constructed a cost model based on data derived from 16 of Manitoba, Canada’s remote satellite units. We included all costs for operation of the unit, transportation, treatment, and capital costs. All costs were presented in 2013 Canadian dollars.

**Results:**

The annual per-patient cost of providing hemodialysis in the satellite units ranged from $80,372 to $215,918 per patient, per year. The median per patient, per year cost was $99,888 (IQR $89,057—$122,640). Primary cost drivers were capital costs related to construction, human resource expenses, and expenses for return to tertiary care centres for health care. Costs related to transport considerably increased estimates in units that required plane or helicopter transfers.

**Conclusions:**

Satellite hemodialysis units in remote areas are more expensive on a per-patient basis than hospital hemodialysis and satellite hemodialysis available in urban areas. In some rural, remote locations, better value for money may reside in local surveillance and prevention programs in addition support for home dialysis therapies over construction of new satellite hemodialysis units.

## Introduction

Chronic Kidney Disease (CKD) and Kidney Failure (KF) requiring kidney replacement therapy with dialysis or transplantation are global health problems with increasing incidence and prevalence[1]. Both CKD and KF are epidemic in Manitoba and other rural and remote northern populations in Canada[2–5], as well as in other remote, rural regions such as the United States[6] or Australia[7]. Over eighty percent of North Americans requiring dialysis receive facility based hemodialysis (FHD), which has serious financial implications for health care systems[8]. FHD is typically delivered three times per week for four hours per treatment and is increasingly becoming decentralized from tertiary care centres in order to increase geographic accessibility for patients.

The term Satellite Hemodialysis (SHD) describes facility based hemodialysis delivered under the remote supervision of a nephrologist who is typically located at a regional tertiary center. Although this model of care was originally described over 35 years ago and has been implemented in many settings[9,10], concern has been expressed that a lack of nephrologist contact in satellite centres may lead to poorer outcomes. The published literature is conflicting on this point. Some studies have reported mortality benefits[11] in association with SHD use, whereas others have suggested a mortality risk[6,12], or have found no impact on mortality[13]. Moreover, SHDs have been associated with improvements in health-related quality of life[14–16], likely related to decreased travel time[17,18] or decreased need for relocation and the associated negative psychosocial consequences[19].

The cost-effectiveness of urban SHD units has been thoroughly described in the available literature[20–23]. However, rural and remote SHD units likely require special attention when considering all component costs associated with providing dialysis. First, these units often function well below physical capacity compared with their urban counterparts. Second, units without a robust infrastructure for attending to moderate to severe illnesses associated with dialysis require costly urgent patient transfers, often by air, to tertiary care centres. Third, human resources are often in short supply in remote centres which demand a premium for compensation.

The primary objective of this study was to evaluate the economic implications of providing SHD in rural and remote locations in comparison to conventional FHD in larger, urban centres from the health care payer’s perspective.

## Materials and Methods

We developed a cost model from the health care payer’s perspective including all direct costs relative to dialysis related care. Data for construction of the cost model was taken from the province of Manitoba, Canada (population >1.2 million, 649,950 km^2^), where the planning and management of all dialysis and CKD related services are provided by the Manitoba Health. Funding is jointly administered by the Manitoba Renal Program (MRP) and regional health authorities. This includes the remote medical management of sixteen satellite hemodialysis units throughout the province called “Local Centre Dialysis Units”. Regional health authorities manage human resources in SHD units for their regions. The study protocol was approved by the University of Manitoba Health Research Ethics Board (HREB). All patients records used in the model development where de-identified prior to analysis.

The cost model is a spreadsheet that summarizes key dialysis related component costs for sixteen SHD units. The operating information of these units was taken using information provided by validated weekly MRP census reports. These reports are provided to clinical and management staff to be used in the planning of dialysis care in the SHD units and include information about each unit’s physical capacity, operating capacity, and the current number of patients dialyzing in each unit.

All costs are presented on an annual, per-patient basis. The upfront capital costs of constructing the units were taken from information provided from MRP financial reports and amortized over a 40 year lifespan, with straight-line depreciation and no assumed salvage value. The capital costs were allotted based on the number of patients treated in each unit. Additionally, annual costs for hemodialysis machines and medical equipment were taken from a previously published study with an assumed useful life of eight years due to a combination of machine wear and technology life cycle[24]. It was assumed that the per-patient costs experienced in the first year of treatment were similar to those experienced in subsequent years[25].

The model included costs applicable to a single-payer Canadian health care system. This system is similar to other single payer health care systems in the US and internationally, in that services are remunerated on a per service basis. Consumables and peripherals costs (e.g. dialyzers, tubing, needles, dialysate), renal medication costs (e.g. erythropoietin, phosphate binders, intravenous iron), dialysis-related laboratory expenses, facility, and hospitalization costs were adapted from a previously published study[24] and assumed to be similar to costs incurred by patients that received treatment in a tertiary centre. An additional increase in the cost of consumables and peripherals was included for shipping supplies to the SHD units from a central tertiary care centre. Staffing costs were determined from MRP financial statements provided for eight of the sixteen evaluated SHD units and calculated separately for communities that were accessible primarily by air travel to accommodate premiums paid to nursing and allied health care staff. Benefits were calculated as a percentage of staffing costs, 18% for non-fly-in communities and 13.5% for fly-in communities due to differences in overtime pay and premiums paid for differences in living arrangements, and were derived from MRP financial statements.

Data used to classify the rate of patients returning from a SHD unit to the tertiary center were identified using the Electronic Kidney Health Record (eKHR) [26] and further retrospective chart review of patients from a representative sample of nine SHD units (3 air and 6 road accessible), assuming that the remaining units would have similar rates of non-acute and urgent returns to their counterparts. The eKHR is the MRP’s electronic database that contains all demographic and hemodialysis patient scheduling data going back to 2011.The eKHR scheduling data has been validated by independent chart review audits and has been the sole source of patient scheduling and location tracking in the Renal Program since 2012. These data sources were used to characterize the number and reasons for an event requiring temporary FHD in an urban tertiary care centre. A period of eight days was required between subsequent return events. Returns longer than three consecutive months were classified as removal from the SHD program. The costs associated with a return to urban tertiary care centres for dialysis included the cost of dialysis in the in-centre tertiary unit, the cost of maintaining the patient’s spot within the SHD program, and the related cost of travel to the tertiary hospital and back to the respective SHD unit. The travel and return costs included ground travel, air travel, ambulance or emergency air flight, escort, hotel, and food costs which are all currently reimbursed for patients by the relevant government agencies. These costs were sourced from aviation service providers in Manitoba for non-acute air returns and Manitoba Health for escort coverage and air ambulance costs.

All costs were inflated and exchanged to 2013 Canadian dollars using the Canadian medical consumer price index[27]. Sensitivity analyses were conducted on all cost drivers using a standard range of +/- 25% based on methodology applied in a previous study[24]. Sensitivity analyses were also conducted by varying the number of patients dialyzing in a unit as this impacted operational efficiency when the number of stations operating was held constant. Further sensitivity analysis was conducted on the rate of returns that resulted in dialysis in urban tertiary centres that were classified as acute.

## Results

The sixteen SHD units ranged in size from four to ten available dialysis stations with operating capacities that varied from 4 to 36 patients based on available staff and stations. The assumed physical capacity of one station operating three shifts a day, six days a week is six patients which is typical utilization for an urban tertiary care hospital. The total number of active patients across the sixteen units during the time period averaged 242 in total and ranged from 2 to 34 patients. Most of the sixteen units did not provide dialysis treatments at full physical capacity. The characteristics of the sixteen units are summarized in Table 1.

**Table 1:** Characteristics of Local Centre Dialysis Units.

Satellite Dialysis Unit	Number of Patients (Dec 31, 2013)	Number of Stations (Dec 31, 2013)	Distance from urban tertiary hospital—km (miles)[11]	Primary Mode of Transport
Unit A	11	4	476 (298)	Road
Unit B	23	7	304 (190)	Road
Unit C	24	10	759 (464)	Air
Unit D	8	4	89 (55)	Road
Unit E	11	6	168 (105)	Road
Unit F	4	4	265 (166)	Air
Unit G	12	6	111 (70)	Road
Unit H	6	4	743 (460)	Air
Unit I	7	4	458 (287)	Air
Unit J	16	10	167 (105)	Road
Unit K	16	6	475 (298)	Air
Unit L	8	6	350 (219)	Road
Unit M	21	7	120 (75)	Road
Unit N	23	6	42 (25)	Road
Unit O	34	10	608 (380)	Air
Unit P	30	9	85 (51)	Road

The annual per patient cost of providing dialysis therapy in the SHD program ranged from $80,372 to $215,918. The median annual per patient cost of dialysis therapy was calculated to be $99,888 with an interquartile range (IQR) of $89,057–$122,640. There was considerable heterogeneity in the cost estimates across units with two of the sixteen units having per patient, per year costs totalling over $200,000 per patient, per year and half of the sixteen units having total per patient, per year costs totalling under $100,000 per patient, per year.

Consumable and peripheral costs were taken from a previous study[24] and assumed to be $6,582 per patient, per year after adjusting for additional costs of shipping estimated at $600 per patient, per year. Renal medications, facility overhead, and hospitalization costs were taken from the literature[24] and totalled, respectively, $7,938, $11,534, and $4,917 per patient, per year. The fees for nephrologist consultation and local centre physician fees totalled $11,778 per year as per provincially determined single-payer fee schedules[28]. The cost attributed to using dialysis services in a tertiary care centre were adapted using a previous cost analysis[24] and varied based on the average number of trips per patient in each respective unit, ranging from $255 to $8,987. These costs are summarized in Tables 2 and 3.

**Table 2:** Cost Model–Average Annual Per-Patient Cost in Communities Accessible by Road.

	Facility HD	Unit N	Unit B	Unit P	Unit G	Unit L	Unit M	Unit E	Unit D	Unit A	Unit J
Dialysis Machinery Costs	$1,551	$2,659	$4,654	$3,723	$3,103	$2,428	$5,319	$9,308	$8,462	$4,654	$4,654
Consumables and Peripherals Expenses	$5,982	$6,582	$6,582	$6,582	$6,582	$6,582	$6,582	$6,582	$6,582	$6,582	$6,582
Human Resource Expenses—Salaries and Wages	$13,380	$19,793	$21,682	$22,888	$19,229	$20,727	$27,271	$19,463	$19,410	$32,832	$34,110
Human Resource Expenses—Benefits	Included	$3,564	$3,905	$4,122	$3,463	$3,732	$4,911	$3,505	$3,495	$5,912	$6,143
Medical Equipment Costs	$423	$678	$743	$785	$1,318	$1,421	$935	$1,334	$1,331	$1,125	$2,339
Renal Medication Expenses	$7,938	$7,938	$7,938	$7,938	$7,938	$7,938	$7,938	$7,938	$7,938	$7,938	$7,938
Dialysis-Related Laboratory Expenses	$1,163	$1,163	$1,163	$1,163	$1,163	$1,163	$1,163	$1,163	$1,163	$1,163	$1,163
Facility Costs	$11,534	$11,534	$11,534	$11,534	$11,534	$11,534	$11,534	$11,534	$11,534	$11,534	$11,534
Capital Costs	N/A	$5,550	$5,614	$4,278	$10,783	$13,008	$6,554	$10,914	$16,327	$13,808	$11,477
Dialysis Transportation Expenses	$1,751	$1,751	$1,751	$1,751	$1,751	$1,751	$1,751	$1,751	$1,751	$1,751	$1,751
Return to Tertiary Care Centre Expenses	N/A	$664	$539	$726	$628	$312	$371	$988	$1,274	$181	$2,085
Costs of Using Dialysis Facility in Tertiary Care Centre	N/A	$1,974	$1,300	$2,521	$2,008	$668	$1,316	$2,678	$3,501	$255	$6,173
Hospitalization-Related Expenses	$4,917	$4,917	$4,917	$4,917	$4,917	$4,917	$4,917	$4,917	$4,917	$4,917	$4,917
Nephrologist and Physician Costs	$7,792	$11,778	$11,778	$11,778	$11,778	$11,778	$11,778	$11,778	$11,778	$11,778	$11,778
Total Annual, Per-Patient Cost	$56,431	$80,545	$84,099	$84,705	$86,195	$87,960	$92,339	$93,854	$99,463	$104,431	$112,642

**Table 3:** Cost Model–Average Annual Per-Patient Cost in Communities Accessible by Air.

	Facility HD	Unit O	Unit H	Unit C	Unit I	Unit F	Unit K
Dialysis Machinery Costs	$1,551	$2,909	$4,296	$3,878	$4,654	$4,072	$4,654
Consumables and Peripherals Expenses	$5,982	$6,582	$6,582	$6,582	$6,582	$6,582	$6,582
Human Resource Expenses—Salaries and Wages	$13,380	$33,208	$34,352	$49,671	$30,667	$63,719	$43,266
Human Resource Expenses—Benefits	Included	$4,492	$4,647	$6,719	$4,148	$8,619	$5,853
Medical Equipment Costs	$423	$772	$1,598	$1,155	$1,426	$2,964	$1,006
Renal Medication Expenses	$7,938	$7,938	$7,938	$7,938	$7,938	$7,938	$7,938
Dialysis-Related Laboratory Expenses	$1,163	$1,163	$1,163	$1,163	$1,163	$1,163	$1,163
Facility Costs	$11,534	$11,534	$11,534	$11,534	$11,534	$11,534	$11,534
Capital Costs	N/A	$3,790	$19,604	$5,669	$17,502	$36,364	$8,231
Dialysis Transportation Expenses	$1,751	$1,751	$1,751	$1,751	$1,751	$1,751	$65,910
Return to Tertiary Care Centre Expenses	N/A	$19,090	$4,827	$23,470	$45,130	$35,293	$37,365
Costs of Using Dialysis Facility in Tertiary Care Centre	N/A	$2,018	$477	$2,918	$7,995	$8,987	$6,689
Hospitalization-Related Expenses	$4,917	$4,917	$4,917	$4,917	$4,917	$4,917	$4,917
Nephrologist and Physician Costs	$7,792	$11,778	$11,778	$11,778	$11,778	$11,778	$11,778
Total Annual, Per-Patient Cost	$56,431	$111,941	$115,463	$139,143	$157,185	$205,681	$216,885

## Influential Model Estimates

**Fig 1:**
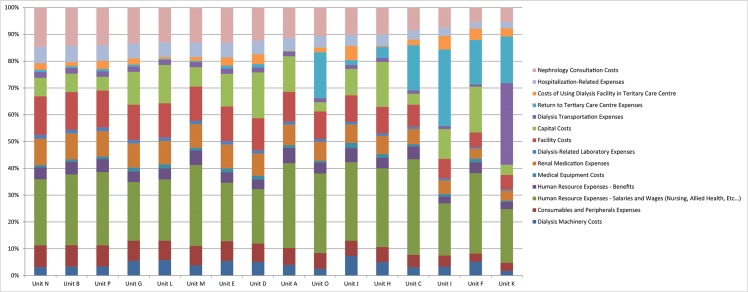
Component Costs as a Percentage of Total Annual, Per-Patient Costs

Component costs in the model that drove the estimates included human resource expenses, capital costs, and transport expenses for routine dialysis and for medical care requiring return to an urban tertiary care centre. Human resource expenses, averaged for communities accessible by ground travel and air travel only, ranged from $19,229 to $63,719 on average per patient, per year. The capital costs of construction for the units ranged from $4,691,712 to $5,507,404 for the five most recent units constructed in 2011 and construction costs did not correlate with the number of available dialysis stations created. After assuming a useful life of 40 years for a newly constructed unit the attributed annualized cost of constructing the unit ranged from $3,790–$36,364 per patient, per year, depending on the number of patients dialyzing in the unit. In a similar fashion, the costs for dialysis machinery ranged from $2,486 to $10,860 per patient, per year. The summary of the impact of each of these cost drivers stratified by SHD unit are presented in Fig 1.

Transport expenses for dialysis were high in the most remote communities where routine treatment often required transport by helicopter due to lack of road access. One outlier unit experienced an estimated annualized per patient cost of $65,910 in helicopter expenses for provision of routine dialysis treatment. Transportation expenses were also influential drivers of the cost estimates in communities that required air transport to and from an urban tertiary hospital for care that could not be delivered in the remote SHD unit or nearby community. For fly-in communities, a baseline rate of 11% was assumed following a retrospective chart review for acute returns requiring emergency air ambulance at a cost of $5250 per one-way trip based on Manitoba Health price quotes. In some of the most remote units patients experienced between 15–22 returns by air to a urban tertiary hospital per year, with attributable costs ranging from $23,470 to $45,130 per patient, per year across these four units. Less remote units in larger road access communities tend to have on site hospitals, which decrease the need for transportation to the larger urban for less severe illness, in addition transportation costs in those communities accessible by road were much lower and ranged from $181 to $2084 per patient per year.

## Sensitivity Analysis

**Fig 2:**
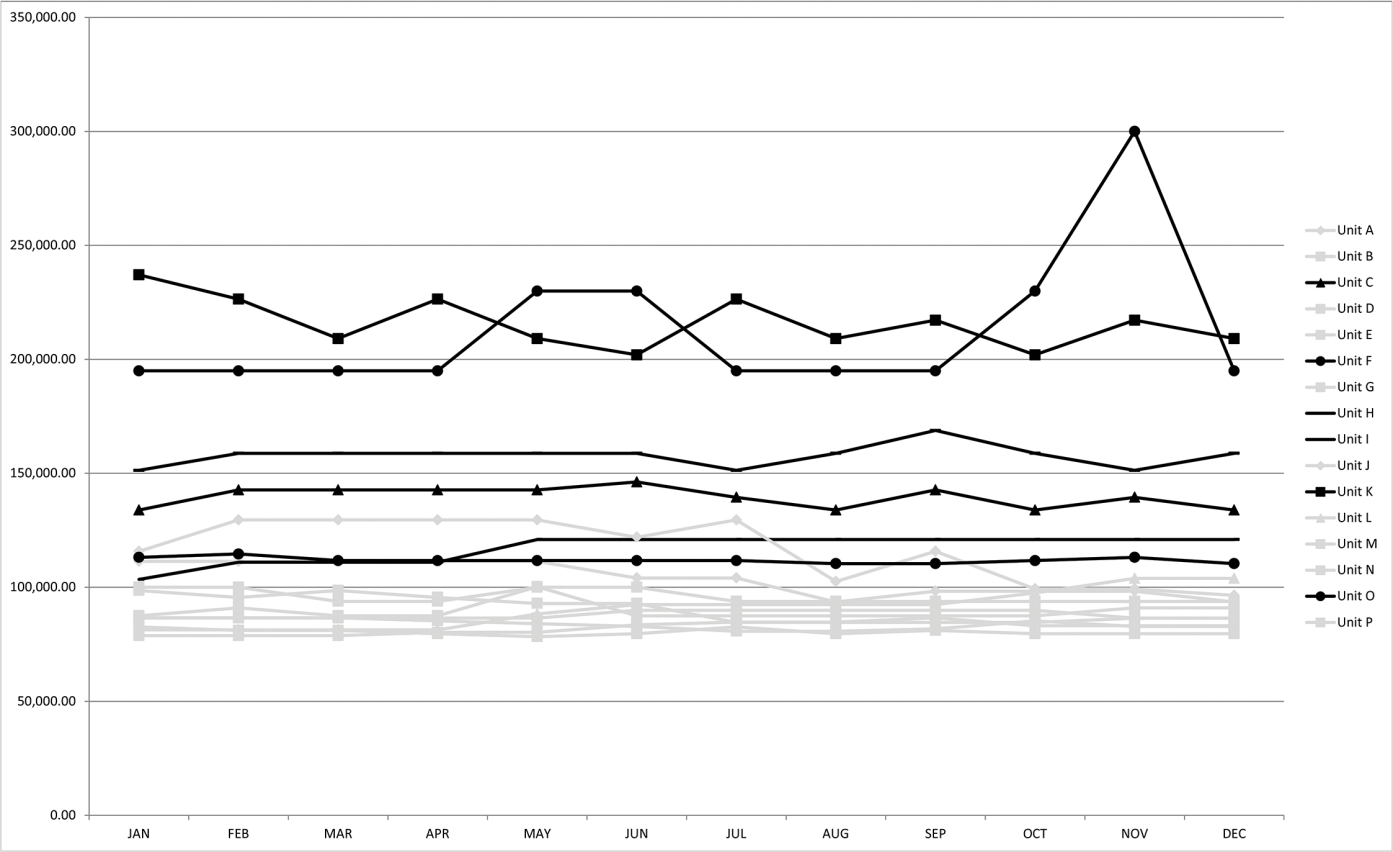
Sensitivity Analysis of Annual, Per-Patient Costs by Monthly Operating Efficiency. Gray lines represent road accessible units and black lines represent primarily air accessible units.

The number of patients dialyzing in each of the sixteen SHD units was not constant throughout the year, and as such, fixed costs attributable to each patient varied with a unit’s occupancy. This effect was notable in units that had a low operating capacity, and particularly in one remote northern unit that temporarily fell to 50% occupancy. If this occupancy was maintained year round, the estimated annual cost of SHD in that unit would reach $299,987 per patient, compared with an annual average of $212,468 based on fluctuations in patient occupancy rates. These changes are illustrated in Fig 2. Furthermore, we considered altering the rate at which patients were transported by ambulance or air ambulance for returns to Winnipeg, Manitoba for care in a tertiary care centre. We varied this from an acute rate of 0 to 22% (baseline 11%). At 0% assumed acute transportations, the estimated per patient costs ranged from $80,155–$208,086, with a median of $101,753 per patient, per year (IQR $88,788–$121,566). At 22% assumed acute transportations, the estimated per patient, per year costs ranged from $80,588–$223,859, with a median of $102,141 per patient, per year (IQR $89,326–$123,714). Raising the costs of human resources and other allied health care expenses by 25% produced a median cost of $106,419 (IQR $93,908–$132,185) across the sixteen units, and decreasing by 25% produced a median cost of $95,417 (IQR $82,431–$111,837). Increasing capital costs attributed to each patient by 25% produced a median cost of $103,655 (IQR $91,718–$126,670) per patient, per year across the sixteen units, and decreasing by 25% produced a median cost of $98,180 (IQR $84,440–$117,677) per year. When we increased or decreased any of the other model component costs it did not produce a meaningful change in overall costs.

## Discussion

In this study we demonstrate the true costs of providing hemodialysis in rural and remote satellite units in the province of Manitoba, Canada. We found that the cost of providing dialysis therapy in this setting could range over four times the cost of providing care in larger, urban settings[24]. Care in some units exceeded $200,000 per patient, per year when optimal operating efficiency was not maintained or with costly travel circumstances. Units that were closer to a major urban centre experienced lower costs for staffing and transportation, and fewer trips to the major urban centre given more advanced medical facilities available in the community. The inability to maintain full utilization of operating capacity in many units was a primary driver of the per-patient specific costs, particularly since the units experienced similar costs of construction despite differences in physical capacity. These findings have health policy implications for rural and remote dialysis units in Canada, the United States, Australia and other countries that may require providing costly renal replacement therapy in areas with low population density and elevated rates of kidney failure[3,29].

A previous study evaluating the costs of SHD discovered that the break-even point for fixed costs is seven patients in a six-station unit[30]. Many of the SHD units we considered did not manage to achieve this threshold. Additionally, this study also found that nursing and physician fees were lower in these SHD units[30]. Our findings showed clear differences between units in proximity to an urban tertiary care hospital and the rural and remote communities, where nurses are often paid a substantial premium to account for differences in living arrangements. In addition to these differences, the difficulties and costs associated with transportation in some northern communities were also substantial cost drivers. One remote unit employed helicopter service multiple times per week to transport patients through difficult terrain with no reliable year-round road access. Moreover, having taken the perspective of the health care payer, the costs described in this model do not include the impact on productivity and caregivers as a result of cumbersome travel requirements.

Although SHD units appear to be cost-effective when constructed in proximity to urban tertiary centres[20–23], the logistical challenges of providing complex care in poorly accessible and sparsely populated locations may considerably alter these cost-effectiveness estimates, as many patients often require treatment from tertiary centers as KF patients are often afflicted with several comorbid conditions[11,31,32]. This would suggest that alternative strategies focused on increasing the prevalence and uptake of home modalities, peritoneal dialysis (PD) and home hemodialysis (HHD), in some rural and remote communities may be economically preferable to SHD. Many studies have shown both PD and HHD to be less costly than in-centre hemodialysis[25,33–37]. However, adoption of these modalities in rural and remote communities may be hampered by poor primary care infrastructure and access[38–40].

The data provided in this cost model are relevant to other regions in Canada in addition to parts of Australia and the United States, where consideration might be given to providing complex care remotely via satellite health centres in rural areas. Despite good health outcomes in Manitoba’s SHD units[11], health outcomes have shown to be worse in remote hemodialysis and peritoneal dialysis patients in other locations in Canada[41], the United States[12], and Australia[7]. As such, the rural units in these sparsely populated areas are often dealing with patients that often need to travel for tertiary care, have high rates of attrition from the dialysis unit due to death, and are faced with the logistical challenges of providing nephrology consultation remotely. We believe these findings would also likely be replicated in other areas where population may be widely dispersed over a large area of land such as Brazil, Russia, or China.

Our study had several important strengths. First, we were able to capture the actual costs of staffing and benefits in many of the units from provided income statements from the Manitoba Renal Program. Second, we were able to obtain complete breakdowns of the capital costs involved in constructing five of the most recent SHD units. Third, we were able to accurately capture the number of times patients returned to larger tertiary care centres for dialysis and other acute care, and thus we were able calculate the incremental costs of travel accrued by patients dialyzing in a SHD unit. Finally, our analysis included and described in detail the characteristics and cost drivers in all 16 geographically and socially diverse SHD units served by the MRP. This granularity of information will be informative for other programs contemplating remote tertiary care in other healthcare settings and regions.

There are also several limitations to this costing model. Microcosting data were not available for shipping costs and an aggregate estimate was used to attribute this item to each of the units; however, although units with required air travel would have higher shipping expenses, this number would likely not be material enough to alter the conclusions of the study as demonstrated by our sensitivity analysis of all component costs. Additionally, these costs cannot be correlated with patient transport expenses for returns to tertiary care centres as the types of urgent medical procedures and comfort level of physicians that are available in each SHD unit influence rates of return in addition to the remoteness of the unit. A further limitation was the lack of information on the severity of the medical condition implied in each return to an urban tertiary care hospital, and therefore estimates were applied to determine the rate of emergency air flight. As such, there may in reality be additional heterogeneity in the costs attributable for transport across the SHD units as less remote units have increased ability to deal with certain medical issues on site due to increased physician presence and availability of diagnostic equipment. Additionally, the cost estimates are sensitive to the volatility in the number of patients receiving dialysis in any given unit. Therefore, units with higher capacity and utilization will have smaller per-patient costs.

In conclusion, there is considerable heterogeneity in the costs associated with providing SHD in rural and remote communities, with some units costing three to four times more than similar care provided in urban settings. The primary factors driving this cost disparity were the costs of air transport in road inaccessible communities and the high capital costs of such units amortized over fewer patients treated. We would expect that these increased health related costs are not limited to the provision of dialysis. Thus, this information may be useful in providing an overview of the factors that affect the costs of operating tertiary care programs in rural and remote regions. Further study should evaluate if better value for money would be achieved by local surveillance and prevention programs in addition to augmenting support for home dialysis modalities as an alternative to the construction of satellite HD facilities in some locations.

## Supporting Information

S1 Dataset All units are de-identified to protect the anonymity of patients receiving dialysis. Information for patient-based measurements presented only in aggregate form.
(XLSX)
